# Alum Adjuvant Enhances Protection against Respiratory Syncytial Virus but Exacerbates Pulmonary Inflammation by Modulating Multiple Innate and Adaptive Immune Cells

**DOI:** 10.1371/journal.pone.0139916

**Published:** 2015-10-15

**Authors:** Ki-Hye Kim, Young-Tae Lee, Hye Suk Hwang, Young-Man Kwon, Yu-Jin Jung, Youri Lee, Jong Seok Lee, Yu-Na Lee, Soojin Park, Sang-Moo Kang

**Affiliations:** 1 Center for Inflammation, Immunity & Infection, Institute for Biomedical Sciences, Georgia State University, Atlanta, GA, United States of America; 2 National Institute of Biological Resources, Incheon, Gyeonggi-do, South Korea; The Ohio State University, UNITED STATES

## Abstract

Respiratory syncytial virus (RSV) is well-known for inducing vaccine-enhanced respiratory disease after vaccination of young children with formalin-inactivated RSV (FI-RSV) in alum formulation. Here, we investigated alum adjuvant effects on protection and disease after FI-RSV immunization with or without alum in comparison with live RSV reinfections. Despite viral clearance, live RSV reinfections caused weight loss and substantial pulmonary inflammation probably due to high levels of RSV specific IFN-γ^+^IL4^-^, IFN-γ^-^TNF-α^+^, IFN-γ^+^TNF-α^-^ effector CD4 and CD8 T cells. Alum adjuvant significantly improved protection as evidenced by effective viral clearance compared to unadjuvanted FI-RSV. However, in contrast to unadjuvanted FI-RSV, alum-adjuvanted FI-RSV (FI-RSV-A) induced severe vaccine-enhanced RSV disease including weight loss, eosinophilia, and lung histopathology. Alum adjuvant in the FI-RSV-A was found to be mainly responsible for inducing high levels of RSV-specific IFN-γ^-^IL4^+^, IFN-γ^-^TNF-α^+^ CD4^+^ T cells, and proinflammatory cytokines IL-6 and IL-4 as well as B220^+^ plasmacytoid and CD4^+^ dendritic cells, and inhibiting the induction of IFN-γ^+^CD8 T cells. This study suggests that alum adjuvant in FI-RSV vaccines increases immunogenicity and viral clearance but also induces atypical T helper CD4^+^ T cells and multiple inflammatory dendritic cell subsets responsible for vaccine-enhanced severe RSV disease.

## Introduction

Respiratory syncytial virus (RSV) is a major human pathogen that causes bronchiolitis in infants and young children as well as serious respiratory illness in the elderly and immunocompromised adults [1, 2]. RSV infection of mice was shown to induce T helper type 1 (Th1) immune responses including IFN-γ, IL-2, and IgG2a isotype antibodies as well as Th2 type immune responses [3, 4]. RSV-specific CD4 T cell responses play a critical role in the clearance of virus and immunopathology [5]. Based on cytokine production profiles, Th1 cells produce IFN-γ, IL-2, and TNF-α whereas Th2 cells produce IL-4, IL-13, IL-6 cytokines associated with inhibiting development of effector CD8 T cell responses [6–13]. Human trials of formalin-inactivated RSV (FI-RSV) formulated with alum adjuvant in 1960s caused vaccine-enhanced respiratory disease resulting in approximately 80% hospitalizations of recipients and two deaths during RSV epidemic winter season [14]. Mice immunized with FI-RSV in alum formulation were shown to have vaccine-enhanced disease and a high ratio of IL-4 to IFN-γ mRNA in lungs after RSV infection, which was diminished by depleting CD4^+^ T cells or IL-4 and IL-10 cytokines [15–17].

Alum adjuvant is widely used in human and animal subunit vaccines. Several studies suggested the potency of alum adjuvant by forming antigen depots in the administration sites and granting the persistence and prolonged release of antigens [18]. Alum preferentially induces Th2 cytokines, which modulate the differentiation of Th2 cells and B cells that generate Th2-associated antibodies (IgG1, IgE) and allergic immune responses [19–22]. Also, alum was shown to raise proinflammtory mediators including IL-1β, CC-chemokine ligand 2 (CCL2; MCP1), CCL11 (eotaxin), histamine and IL-5 as well as neutrophils, eosinophils, inflammatory monocytes, myeloid dendritic cells (DCs), and plasmacytoid DCs [23, 24].

DCs connecting innate and adaptive immunity play an important role in protection and immunopathology. DCs are divided into multiple subsets including conventional CD11b^+^, CD103^+^, and B220^+^ plasmacytoid dendritic cells based on their phenotypes in the lung as well as into lymph node-resident CD4^+^CD8^-^, CD4^-^CD8^+^, CD4^-^CD8^-^ DCs [25, 26]. Such DC subsets have been suggested to be programmed to direct the differentiation of CD4 T cells into either IFN-γ-secreting Th1 cells or IL-4-secreting Th2 cells [27, 28]. CD11b^+^ DCs are effective in activating effector CD4 T cells whereas CD103^+^ DCs prime naïve CD8 T cells [29]. Plasmacytoid DCs (pDCs) were shown to be important for inducing antiviral immunity through IFN-α production and enhancing CD8 T cell responses during RSV infection [30, 31]. Other studies demonstrated that pDCs contribute to severe RSV disease and elevated mortality during lethal influenza virus infection [32, 33]. Formalin inactivation of RSV has been considered a major factor responsible for inducing FI-RSV vaccination-enhanced respiratory disease probably due to poor induction of neutralizing antibodies [34–37]. However, possible effects of alum adjuvant on FI-RSV vaccine-enhanced RSV disease, effector T cell responses, and mobilization of DC subtypes have not been well understood yet despite its common use in human vaccines.

In this study, we have determined the roles of alum adjuvant in inducing humoral and cellular immune components contributing to immunogenicity, protection, and disease after FI-RSV vaccination and then RSV infection. We found that alum in FI-RSV (FI-RSV-A) significantly contributed to enhancing RSV-specific IgG1 isotype antibody responses and clearing lung viral loads. Nonetheless, FI-RSV-A immune mice showed significant body weight loss and vaccine-enhanced disease compared to unadjuvanted FI-RSV (FI-RSV) immune mice. Alum in FI-RSV was found to be responsible for inducing eosinophilia, mucus production, and lung histopathology by increasing RSV specific IL-4^+^ an TNF-α^+^ Th2 CD4^+^ T cell responses, and the mobilization of multiple DC subsets including CD11b^+^, CD103^+^, pDCs, and CD4^+^ DCs.

## Materials and Methods

### Mice and virus

Six to eight week-old female BALB/c or C57BL/6 (B6) wild type mice were purchased from Charles River Laboratories International (Wilmington, MA) and Jackson Laboratory (Bar Harbor, Maine) respectively. All animal studies were conducted according to the guidelines of Georgia State University Institutional Animal Care and Use Committee (IACUC). RSV A2 strain was originally obtained from Dr. Barney Graham and prepared as described previously [38].

### Cells, antibodies, and Reagents

HEp-2 cells were purchased from the American Type Culture Collection (ATCC, Rockville, MD, USA) and maintained in Dulbecco’s modified Eagle’s medium (DMEM; GIBCO-BRL, Grand Island, NY) with 10% fetal bovine serum (FBS, GIBCO-BRL), 2 mM glutamine, penicillin and streptomycin (GIBCO-BRL) at 37°C with 5% CO_2_. Mouse anti-RSV F monoclonal antibody (131-2A) were purchased from Millipore (Billerica, MA, USA). The horseradish peroxidase (HRP) conjugated goat anti-mouse immunoglobulin G (IgG), IgG1, and IgG2a were from Southern Biotech (Birmingham, AL, USA). Aluminum hydroxide (Alum) was purchased from Sigma-Aldrich (St. Louis, MO).

### Preparation of formalin-inactivated RSV (FI-RSV) in alum adjuvant

RSV and FI-RSV were prepared in HEp2 cells by modification of the previously reported method [39, 40]. Briefly, clarified RSV by centrifugation and filtering was inactivated with formalin (1:4000 vol/vol) for 72 h at 37°C. FI-RSV was adsorbed to aluminum hydroxide (4mg/ml) for FI-RSV alum adjuvant vaccine formulation.

### Immunization, RSV challenge, and sample collection

Female BALB/c mice (5 per groups) were immunized intranasally (i.n.) with 5 μg of FI-RSV (approximately equivalent to 1x10^6^ PFU) with or without alum (20 μg or 5 μg alum in 100 μl FI-RSV per mouse), PBS (naïve control) or infected i.n. with live RSV (1x10^6^ PFU) twice using a prime-boost protocol at a 4-week interval. Blood samples were collected at 3 weeks after prime and boost immunization. Unimmunized naive, immunized, or RSV re-infected mice were i.n. challenged with RSV A2 (2x10^6^ PFU per mouse) under isoflurane anesthesia at 12 weeks after boost immunization and body weight changes were monitored. Mice were scrificed by carbon dioxide inhalation and a cervical dislocation. Individual samples such as lung, mediastinal lymph node (MLN), spleen, bone marrow, and bronchoalveolar lavage fluids (BALF) were collected at 5 days post-challenge (d.p.c.). Results described in this study were derived from two independent sets of experiments. All animal studies were conducted according to the guidelines of Georgia State University Institutional Animal Care and Use Committee (IACUC). Georgia State University IACUC specifically approved this study. The approved IACUC protocol number is A14025.

### ELISA assay for antibody and cytokines responses

Virus and F or G protein-specific antibody isotypes (IgG, IgG1, and IgG2a) were determined in samples by enzyme-linked immunosorbent assay (ELISA) as previously described [38, 41]. Briefly, purified live or formalin-inactivated RSV (4 μg/ml) and purified recombinant F (BEI, NIH) or G protein (100 ng/ml, Sino biological, North Wales, PA) were used as a coating antigen. The antibody responses were detected using the secondary antibodies of HRP-conjugated goat anti-mouse IgG, IgG1, and IgG2a (Southern Biotechnology). Antibody concentrations were quantified using the standard curve for each IgG isotype antibodies. The levels of interleukin-4 (IL-4) and IL-6 (eBioscience, SanDiego, CA) and chemokine eotaxin (R&D Systems, Minneapolis, MN) in lung homogenates were measured using ELISA kits according to the manufacturer’s instructions.

### RSV immuno-plaque assay

Viral titers in individual lung samples and neutralizing antibody titers in immune sera were determined using an immuno-plaque assay (IPA) as previously described [38] and [41]. Briefly, equal volumes of diluted heat-inactivted sera were mixed with 200 PFU/well of RSV A2 and the immune mixtures or RSV only were incubated for 1 h at 37°C, 5% CO_2_. The serially diluted lung homogenates as well as the immune mixtures or virus only were added into monolayer HEp-2 cells and were adsorbed for 1–2 h then overlaid with growth medium containing 0.8% low gelling temperature agarose prior to incubation for 3 days. After fixation in formalin, the plaques were detected using the anti-RSV F monoclonal antibody. The viral titer detection limit is approximately 40 PFU from lung sample of mouse in this assay.

### ELISpot assay

Splenocytes, lung, and bone marrow cells were isolated from tissue samples at 5 d.p.c.. RSV F-specific antibody secreting cells were determined on Multi-screen 96 well plates (Millipore, Billerica, MA) pre-coated with purified recombinant F protein (400ng/ml) by enzyme-linked immunospot (ELISpot) assay as previously described [42]. The spots of anti-F secreting cells were developed with biotinylated anti-mouse IgG and streptavidin-AP (Southern) and 3,3’-diaminobenzidine tetrahydrochloride (DAB, Thermo Scientific). The spots of IFN-γ and IL-4 secreting cells were determined on Multi-screen 96 well plates coated with cytokine specific capture antibodies as described [40–42]. Briefly, lung cells (0.2x10^6^) per well were cultured with peptide F_92-106_ (ELQLLMQSTPATNNR, 4 μg/ml), F_85-93_ (KYKNAVTEL), G_183-195_ (WAICKRIPNKKPG), and M2_82-90_ (SYIGSINNI) as previously described [43, 44]. After 36 h stimulation, the spots were developed with biotinylated mouse IFN-γ or IL-4 antibody, alkaline phosphatase labeled streptavidin (BD Pharmingen) and then counted using an ELISpot reader (BioSys, Miami, FL).

### Flow cytometry

BAL fluids were obtained by infusing 1 mL of PBS into the lungs via the trachea using a 25-gauge catheter (Exelint International Co., Los Angeles, CA) at 5 d.p.c. and 7 days post immunization (d.p.i) before challenge infection. Lung cells were isolated by homogenization of tissue through 70 μm cell strainers and Percoll gradient (44% and 67%) centrifugation. For detection of intracellular cytokine-secreting cells, lung cells were stimulated with F_92-106_, F_85-93_, G_183-195_, and M2_82-90_ peptide. Intracellular FACS staining was performed using a cytofix/cytoperm kit (BD Biosciences) according to manufacturer’s instructions. Cells were stained with antibodies for intracellular cytokines and surface markers including IFN-γ, IL-4 (eBioscience), TNF-α (BioLegend), CD45, CD3, CD4, CD8, Siglec F (BD Biosciences). To determine memory B cells, plasma cells, and DC phenotypes, mediastenial lymph node (MLN), spleen, and bone marrow cells were isolated from mice at 5 d.p.c. and 7 d.p.i.. Cells were stained with CD19, IgD, F4/80, CD11b, CD11c, and PNA or GL7 (eBioscience) as well as with CD45R/B220, CD138, and CD103 (BioLegend). The PNA^+^ or GL7^+^ germinal center B cells and CD138^+^ plasma cells were analyzed from pre-gated mature B cells (CD19^+^IgD^-^B220^+^/CD4^-^CD8^-^ and CD19^+^IgD^-^B220^int^/CD4^-^CD8^-^) in spleens and MLN. The DC populations of B220^+^, CD103^+^ and CD11b^+^ in MLN and lungs were determined from pre-gated CD11c^+^F4/80^-^ cells. Stained cells were acquired on a FACSCanto flow cytometer (BD), and analyzed using Flow Jo software (Tree Star Inc., Ashland, OR).

### Lung histopathology

The lungs collected at 5 d.p.c. and 7 d.p.i. were fixed with 10% formalin in PBS, embedded in paraffin blocks. Lung tissue sections were stained with hematoxylin and eosin (H&E) and periodic acid-schiff stain (PAS) to assess histologic changes and airway mucin expression respectively as described [45]. The sections were examined to score the degree of inflammation in interstitrum or alveolar spaces as described [46, 47]. PAS-positive spots were detected in 25 randomly selected area in the airway epithelium.

### Statistical analysis

The data obtained using the mean and the standard error of means (SEM) value from two independent experiments and 5 mice per group. The statistical analyzes were performed by either a one-way ANOVA with Tukey multiple comparison test or two-way ANOVA in GraphPad Prism version 5 (GraphPad software Inc, San Diego, CA). A *p* value less than 0.05 was considered significant.

## Results

### Alum significantly enhances RSV specific antibody responses and neutralizing activity

**Fig 1:**
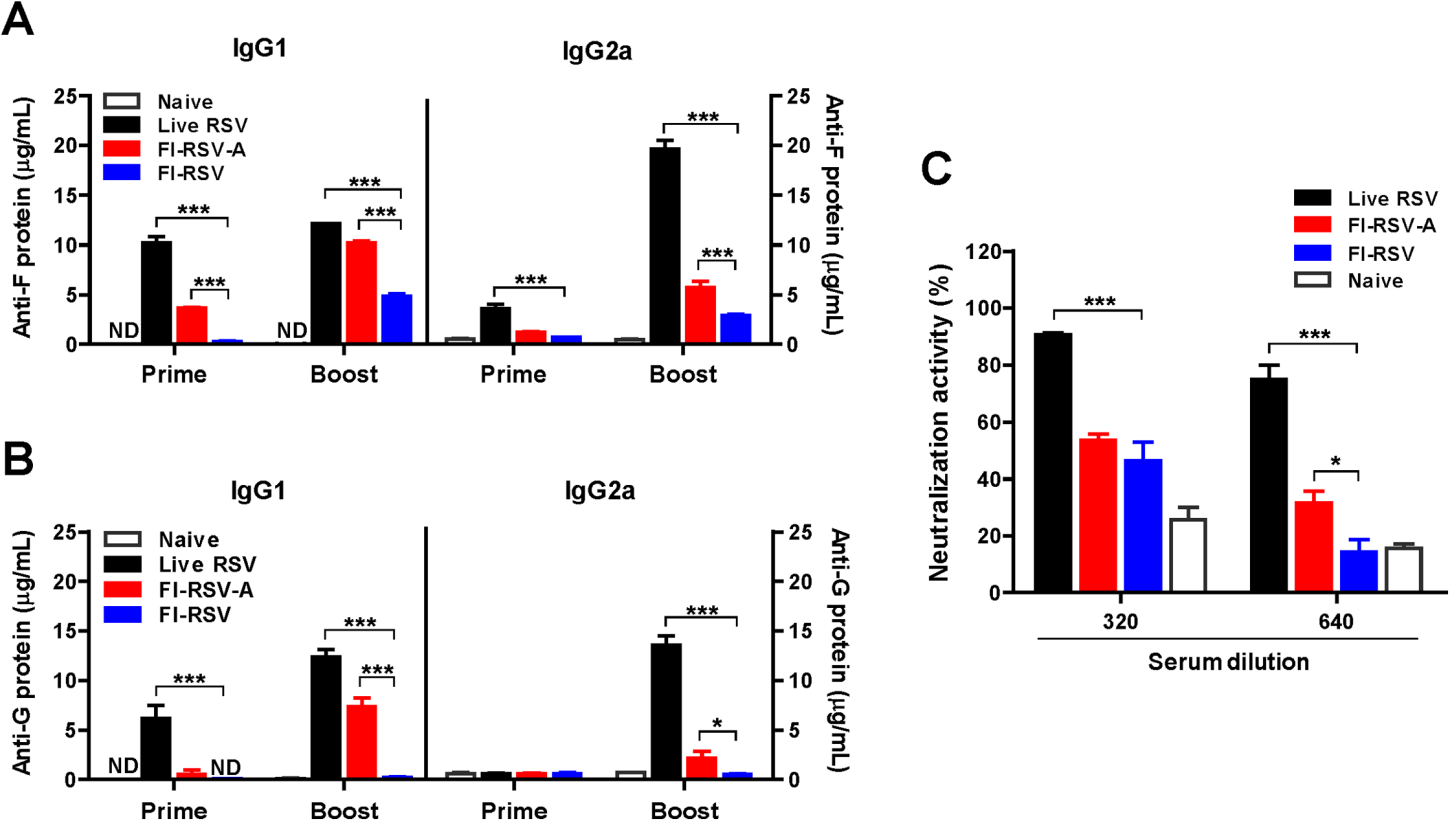
Alum adjuvant in FI-RSV enhances RSV specific antibody responses and neutralizing activity. The levels of isotype IgG antibodies specific for RSV F and G protein were measured in serum samples at 3 weeks after prime and boost administration (n = 5). (A) RSV F specific IgG1 and IgG2a isotype antibodies. (B) RSV G specific IgG1 and IgG2a isotype antibodies. (C) RSV neutralizing activity. Naïve: unimmunized mice, live RSV: re-infection of mice with RSV A2 (1x10^6^ PFU), FI-RSV-A: mice with i.n. immunization with FI-RSV in the alum adjuvant formulation, FI-RSV: mice with i.n. immunization with FI-RSV without alum. Results are presented as mean ± SEM. Statistical significances were performed by two-way ANOVA in GraphPad Prism; *** *p*<0.001, * *p*<0.05. ND: Not detected.

The underlying mechanisms by which FI-RSV-A immunization induces vaccine-enhanced RSV disease have not been well understood. RSV F and G proteins are the major targets for neutralizing antibodies. We investigated the effects of alum adjuvant on the immunogenicity of FI-RSV after intranasal immunization of mice with alum-adjuvanted FI-RSV (FI-RSV-A) in comparison with unadjuvanted FI-RSV (FI-RSV) and live RSV reinfections (live RSV). First, we evaluated RSV F and G-specific antibody responses (Fig 1). Live RSV-reinfected mice showed highest levels of IgG2a isotype antibodies specific for RSV F and G. Boost IgG1 antibodies in the FI-RSV-A group were comparable to those in the live RSV group whereas IgG2a isotype antibodies were lower than those in the live RSV group (Fig 1A and 1B). Prime immunization with FI-RSV did not induce RSV specific antibodies at detectable levels and boost immunization with FI-RSV induced RSV F (but not RSV G) specific antibodies at low levels (FI-RSV, Fig 1A and 1B). FI-RSV-A immune mice showed higher levels of IgG1 and IgG2a isotype antibodies specific for RSV F in boost sera compared with those of FI-RSV immune mice (Fig 1A and 1B).

We further analyzed the levels of RSV-specific antibody responses in immune sera using whole live RSV or FI-RSV as an ELISA coating antigen (Table 1). As shown in Table 1, mice with live RSV reinfections showed significantly higher levels of serum IgG2a antibodies compared with those in FI-RSV-A and FI-RSV immune mice. As expected, FI-RSV-A immune mice showed higher levels of IgG1 antibodies in boost sera than those of FI-RSV immune mice (Table 1).

**Table 1:** The levels of antibodies to purified live and formalin inactivated RSV in immune sera. The levels of IgG isotype and IgA antibodies specific for purified live RSV and FI-RSV were measured in serum samples at 3 weeks after prime and boost administration.

RSV-specific antibodies (ng/mL)							
	IgG		IgG1		IgG2a		IgA
Group	Prime	Boost	Prime	Boost	Prime	Boost	Boost
PBS	243±00	347±00	56±03	104±01	240±02	272±02	8±00
Live RSV	5,954±21	7,738±33	2,536±26	3,536±22	4,615±17	8,518±13	338±00
FI-RSV alum	2,712±19	5,995±39	1,389±24	3,197±01	551±05	1,819±18	163±01
FI-RSV	1,054±03	4,068±16	257±01	1,974±04	340±02	1,923±05	200±00
Formalin inactivated RSV-specific antibodies (ng/mL)							
	IgG		IgG1		IgG2a		IgA
Group	Prime	Boost	Prime	Boost	Prime	Boost	Boost
PBS	184±02	224±08	31±00	50±00	181±03	233±01	18±00
Live RSV	5,731±11	7,269±08	3,130±07	3,512±07	2,301±08	8,225±33	240±11
FI-RSV alum	1,993±02	6,245±28	1,520±22	3,060±17	681±01	3,284±99	86±03
FI-RSV	708±04	2,498±19	162±01	1,381±06	335±03	1,313±02	28±00

Neutralizing antibody is an important parameter in protective efficacy against RSV infection. We determined neutralizing activity against RSV in boost immune sera using a plaque reduction assay (Fig 1C). The neutralizing titers of FI-RSV-A immune sera were approximately 54% and 31% in 320 and 640 fold dilutions respectively. The FI-RSV-A group showed higher neutralizing activity compared to that of FI-RSV immune mice that showed approximately 14% of plaque reduction with 640-fold diluted sera (*p*<0.05, Fig 1C). As expected, live RSV immune sera showed highest neutralizing activity of 90% and 75% in 320 and 640 fold-diluted sera (Fig 1C). These results suggest that alum adjuvant in FI-RSV vaccines significantly enhances RSV specific binding and neutralizing antibody responses.

### Alum in FI-RSV is an effective adjuvant in clearing lung viral loads but leads to severe weight loss

**Fig 2:**
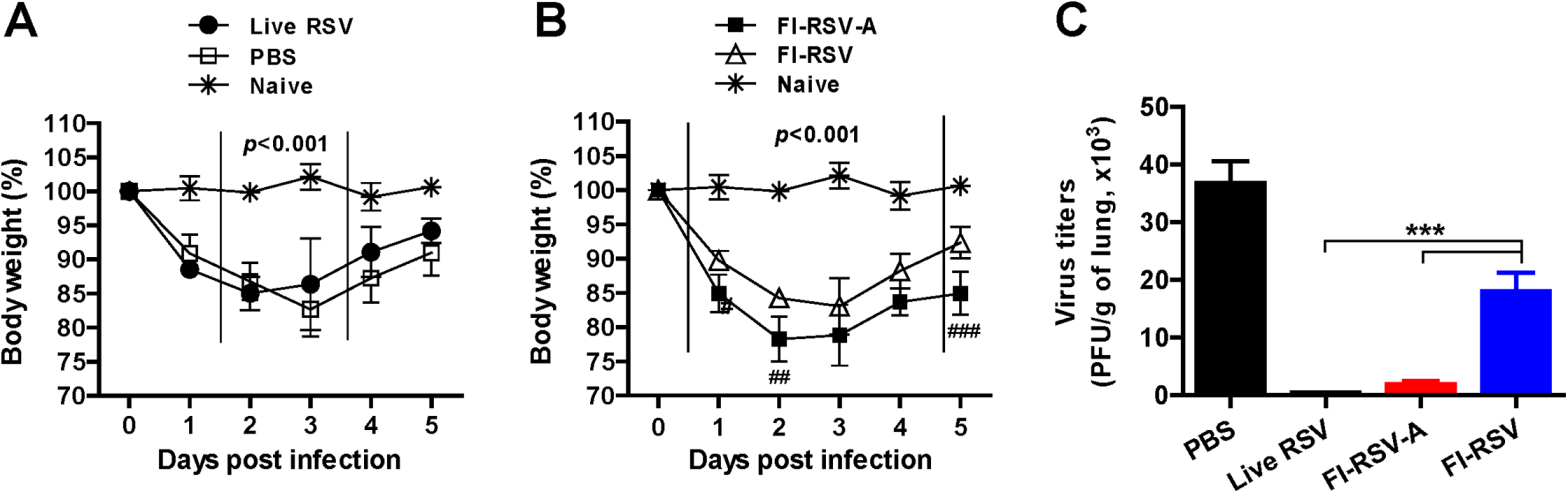
Alum in FI-RSV enhances lung viral clearance but leads to severe weight loss. At 12 weeks post-boost immunization, immune and PBS mock control mice (n = 5 per group) were i.n. challenged with 2x10^6^ PFU RSV A2. Naïve: unimmunized mice without RSV infection, PBS: PBS mock control mice with RSV infection, live RSV: live RSV re-infection mice after RSV infection, FI-RSV-A: FI-RSV-A immune mice after RSV challenge, FI-RSV: FI-RSV immune mice after RSV challenge. (A) Body weight changes in naïve and prior live RSV reinfected mice after RSV challenge. (B) Body weight changes in naïve, FI-RSV-A, and FI-RSV mice after RSV challenge. (C) RSV titers in lungs. After 5 d.p.c., individual lungs were collected and virus titers were determined. Results are presented as mean ± SEM (n = 5). Statistical significances were performed by two- or one-way ANOVA and Tukey’s multiple comparison tests in GraphPad Prism; (A and B) *p*<0.001, compared with naïve control and immunized or RSV reinfection group; (B) ^##^ *p*<0.01, ^###^ *p*<0.001 between with FI-RSV-A and FI-RSV.

Mice were challenged with RSV at 12 weeks after boost immunization and weight changes of mice were monitored (Fig 2). Mice with live RSV reinfections showed approximately 15% of weight loss at 2 d.p.c., and then recovered faster than RSV primary infected naïve mice (Live RSV, Fig 2A). FI-RSV-A immune mice exhibited 20 to 25% of severe weight loss at 2 to 3 d.p.c., whereas FI-RSV immune mice displayed approximately 17% of weight loss at 3 d.p.c. (FI-RSV-A versus FI-RSV, Fig 2B).

Mock (PBS) treated naïve mice or FI-RSV immune mice were found to have highest viral loads in the lungs among the groups. The FI-RSV-A group showed significantly lower levels of RSV lung titers than those in the FI-RSV group (FI-RSV-A vs. FI-RSV, Fig 2C). FI-RSV immune mice showed lower RSV titers than naïve mice (PBS versus FI-RSV, Fig 2C). Mice with prior RSV reinfections almost cleared lung viral loads at 5 d.p.c. (Fig 2C). These results suggest that alum in FI-RSV is an effective adjuvant in improving pulmonary viral clearance but causes more severe weight loss compared to FI-RSV.

### Alum in FI-RSV contributes to inducing antibody-secreting plasma and germinal center B cells

**Fig 3:**
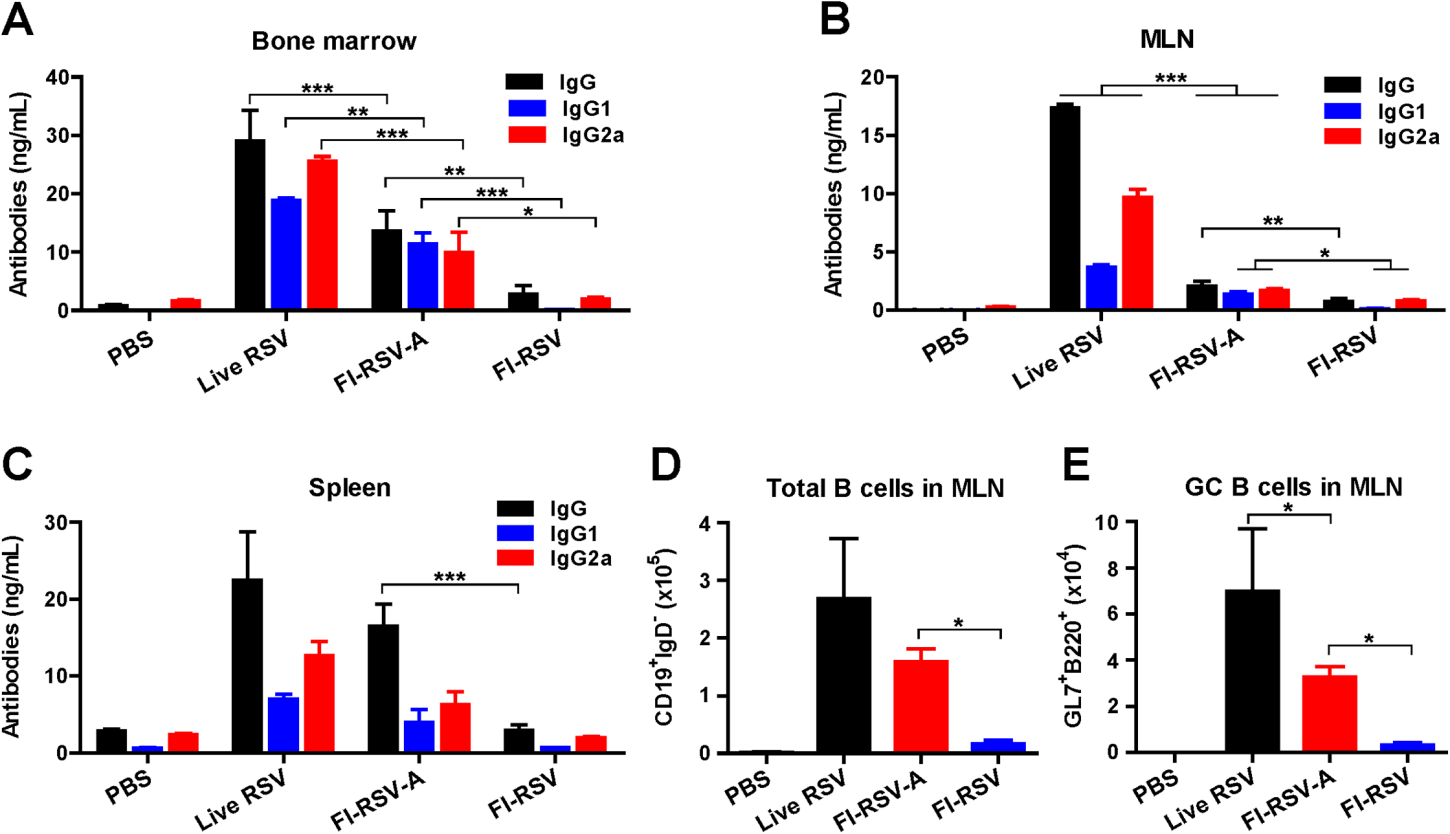
Antibody-secreting plasma and germinal center B cell responses before challenge. Bone marrow, splenocytes, and MLN (n = 5) were collected at 7 days post boost immunization (d.p.i.). MLN cells were stained with surface markers to germinal center (GC) B cells (GL7^+^B220^+^) by flow cytometry. (A) RSV-specific IgG (IgG, IgG1, IgG2a) antibody secreting cell responses in bone marrow after 1 day *in vitro* cultures. (B) RSV specific antibody secreting cell responses in MLN after 5 day *in vitro* cultures. (C) RSV specific antibody secreting cell responses in spleens after 5 day *in vitro* cultures. (D) Total B cells (CD19^+^IgD^-^) at 7 d.p.i.. (E) GC B cells (GL7^+^B220^+^) at 7 d.p.i.. Groups are the same as described in the Fig 1. Results are presented as mean ± SEM (n = 5). Statistical significances were performed by two- or one-way ANOVA and Tukey’s multiple comparison tests in GraphPad Prism; *** *p*<0.001, ** *p*<0.01, * *p*<0.05.

To assess antibody-secreting plasma and germinal center (GC) B cell responses, the bone marrow, lymph nodes, and spleen cells were collected 7 days post boost immunization for flow cytometry and ELISpot analysis (Fig 3A–3E). The live RSV reinfection group showed highest levels of F specific antibody (IgG, IgG1, IgG2a) secreting cell responses in bone marrow, lymph nodes, and spleen (Fig 3A, 3B and 3C). Alum adjuvant in FI-RSV (FI-RSV-A) resulted in enhancing IgG isotype particularly IgG1 antibody secreting cell responses in bone marrow, lymph nodes, and spleens compared to the FI-RSV alone group which induced lowest antibody secreting cell responses before challenge A (Fig 3A–3C). A similar pattern of total B cells, GC phenotypic (PNA^+^B220^+^CD138^-^CD19^+^IgD^-^) B cells was observed among groups before challenge (Fig 3D and 3E).

**Fig 4:**
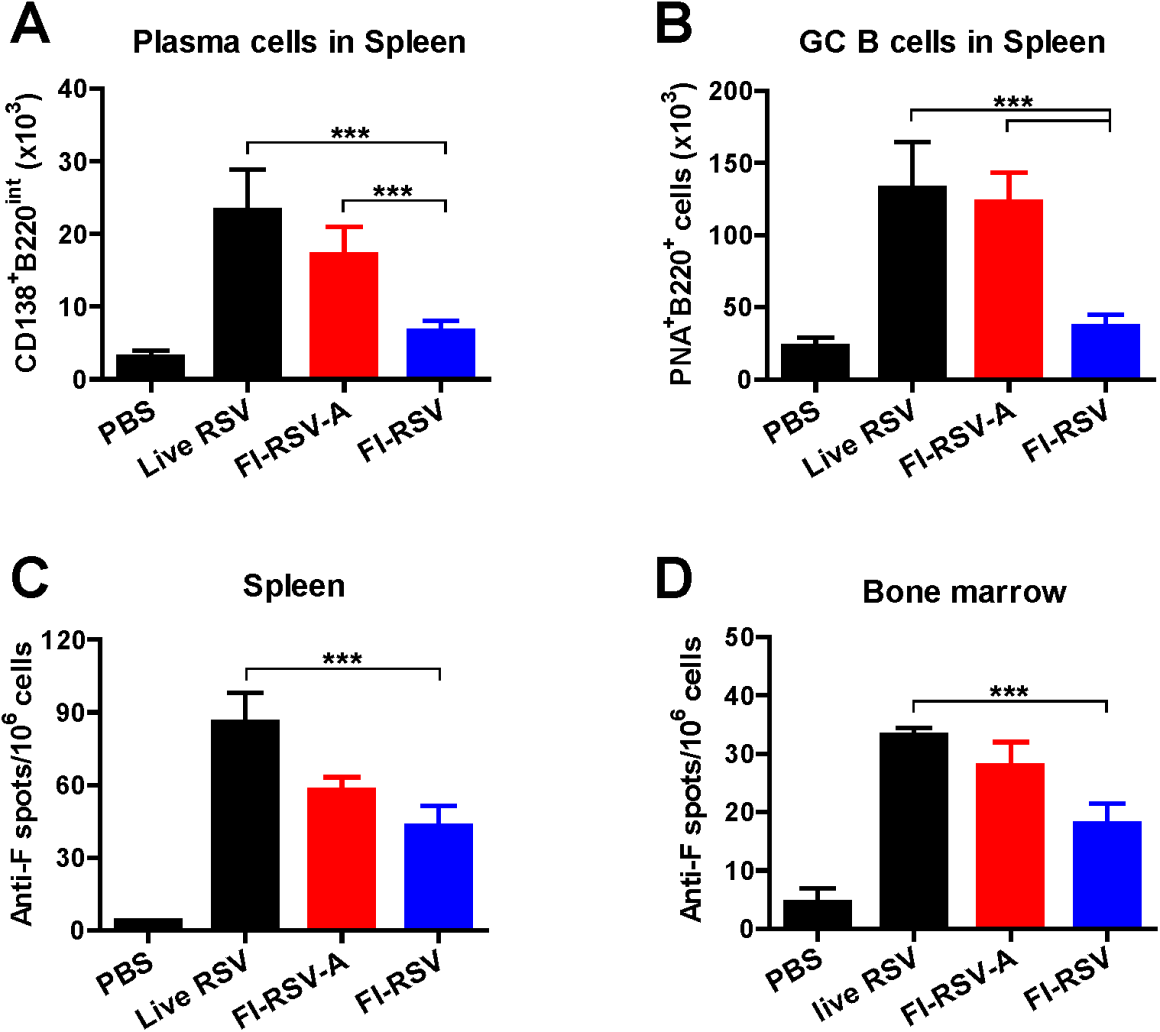
RSV F-specific antibody secreting cell responses, and plasma and germinal center B cells after challenge. Bone marrow and splenocytes (n = 5) were collected at 5 d.p.c. and splenocytes and MLN cells were stained with surface markers to assess plasma and germinal center (GC) B cells by flow cytometry. CD138^+^B220^int^PNA^-^ phenotypic plasma cells (A) and PNA^+^B220^+^CD138^-^ phenotypic GC B cells (B) in spleens at 5 d.p.c.. (C and D) RSV F specific antibody secreting cell spots in spleen (5 day) and bone marrow cells (1 day) after *in vitro* culture at 5 d.p.c.. Results are presented as mean ± SEM (n = 5). Statistical significances were performed by two- or one-way ANOVA and Tukey’s multiple comparison tests in GraphPad Prism; *** *p*<0.001, ** *p*<0.01.

At 12 weeks after boost, we also determined B cell responses day 5 post challenge (Fig 4). Significantly higher levels of CD138^+^B220^int^CD19^+^IgD^-^ phenotypic plasma cells (*p*<0.001) and GC phenotypic B cells (*p*<0.001) were observed in spleens of live RSV and FI-RSV-A immune mice compared to those in FI-RSV immune mice (Fig 4A and 4B). Live RSV reinfection induced highest numbers of RSV F specific antibody secreting cell spots in spleens and bone marrow (Fig 4C and 4D). FI-RSV-A immune mice showed higher levels of RSV F-specific antibody secreting cell spots in spleen and bone marrow samples than those in FI-RSV immune mice (Fig 4C and 4D). These results indicate that alum in FI-RSV vaccines contributes to enhancing RSV F-specific plasma and memory B cell responses.

### Alum in FI-RSV contributes to severe pulmonary inflammation, mucus production, and eosinophilia

**Fig 5:**
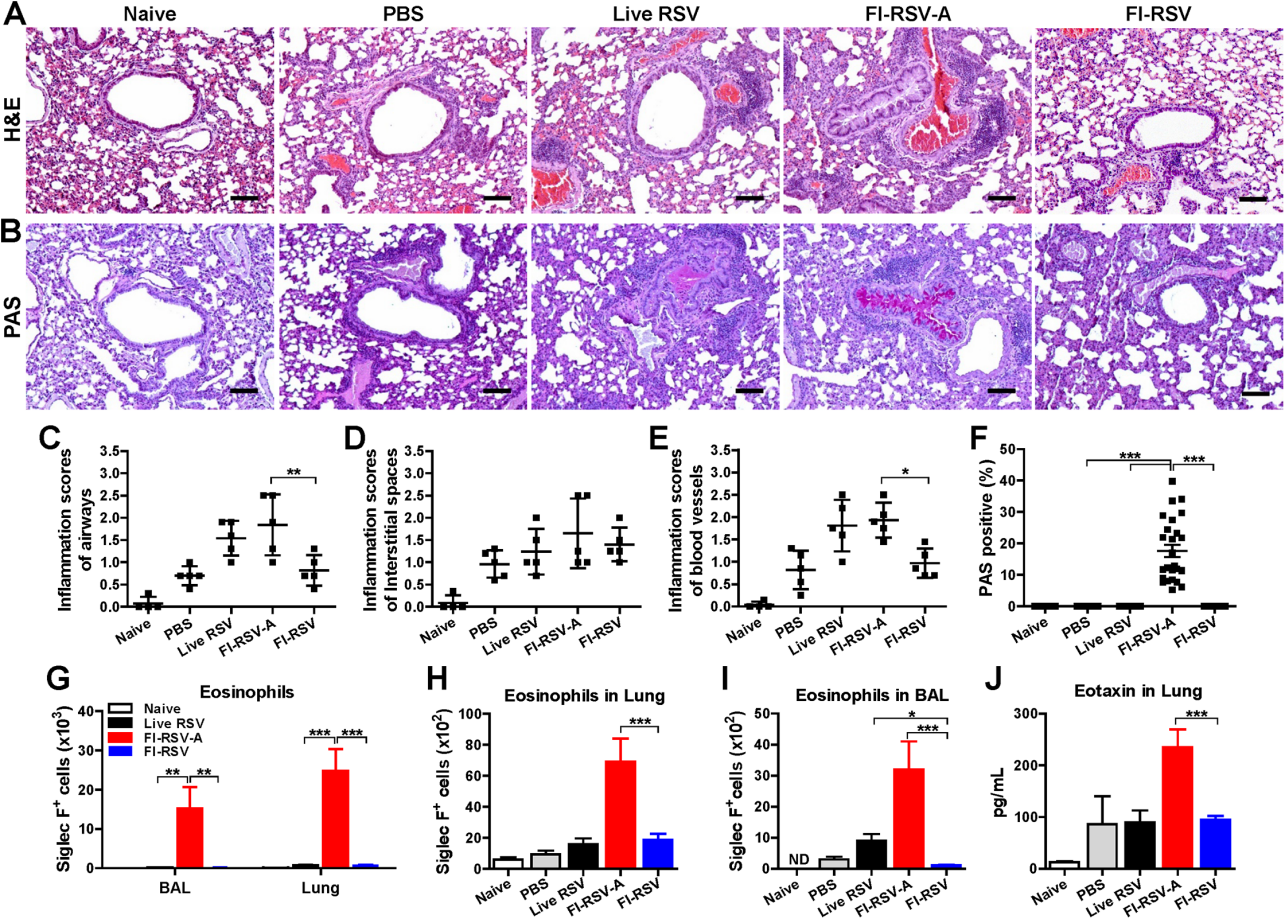
Alum in FI-RSV enhances inflammation, mucus production, and eosinophilia. (A-F) Lung tissues collected from individual mice (n = 5 per groups) at 5 d.p.c. were stained with Hematoxylin and Eosin (H&E) and Periodic-Schiff (PAS) to assess peribronchiolar, alveolar pneumonia, and mucus production. (A) Photomicrographs of H&E. Scale bars indicate 100 μm for 100×. (B) Photomicrographs of PAS. H&E stained tissue sections were scored in the airways (C), interstitial spaces (D), and blood vessels (E) for quantitative comparison of pulmonary inflammation and histopathology on a scale of 0 to 3 as diagnostic criteria. (F) PAS positive airway mucus production. Airway mucus production was scored as percentages of 5 individual airways in each mouse. (G) Siglec F^+^ eosinophils at 7 d.p.i. before challenge. Siglec F^+^ eosinophils were detected in BAL (bronchoalveolar lavage) cells and lungs from gated granulocytes (CD11b^+^CD11c^-^F4/80^+^CD45^+^) by flow cytometry. Siglec F^+^ eosinophils in lungs (H) and BAL cells (I) at 5 d.p.c.. (J) Eotaxin production was determined in lung homogenates collected at 5 d.p.c. by ELISA. Results are presented as mean ± SEM. Statistical analysis were analyzed by two- or one-way ANOVA and Tukey’s multiple comparison test in GraphPad Prism; *** *p*<0.001, ** *p*<0.01, * *p*<0.05. ND: Not detected.

Lung tissue sections were stained with H&E at 5 d.p.c. to evaluate pulmonary histopathology. FI-RSV-A immune mice showed most severe inflammation around the airways, blood vessels, and interstitial spaces after RSV challenge (Fig 5A and 5C–5E). Live RSV reinfections also induced substantially high inflammation around the airways, interstitial spaces, and blood vessels, which are similar to those observed in the FI-RSV-A group (Live RSV versus FI-RSV-A, Fig 5A and 5C–5E). In contrast, FI-RSV immune mice did not induce such high inflammation, similar to mock control (PBS) mice upon RSV infection (PBS versus FI-RSV, Fig 5A and 5C–5E). Interestingly, FI-RSV-A and live RSV reinfection group showed significant pulmonary inflammation at day 7 after boost immunization or live RSV re-infection (Fig A panels A-D in S1 File).

**Fig 6:**
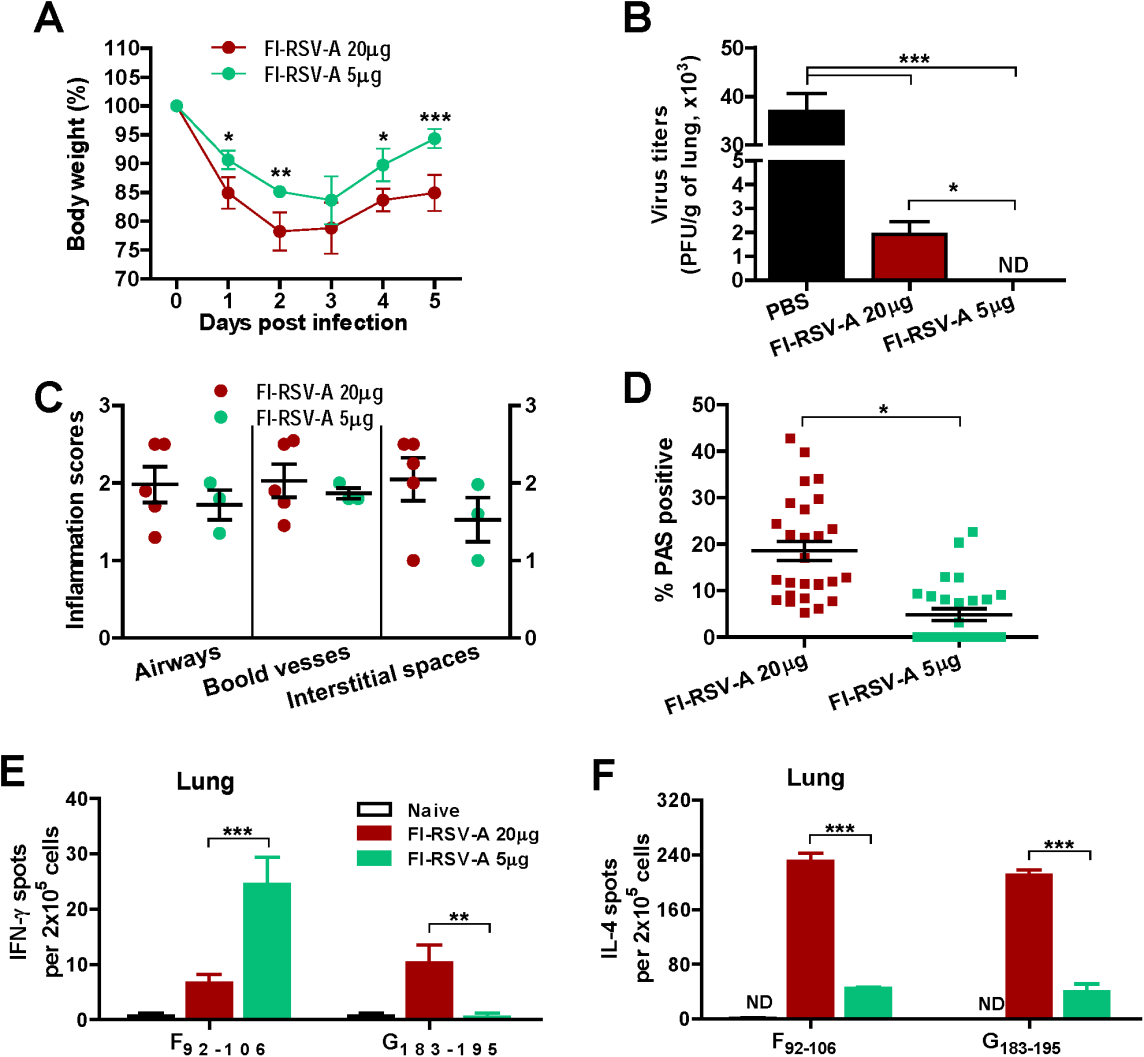
Effects of a low dose alum adjuvant in FI-RSV on body weight, protection, lung inflammation and effector cells. A low dose of alum (5 μg) adjuvant effects was compared with FI-RSV-A (with 20 μg alum) after FI-RSV vaccination and RSV challenge of mice (n = 5). (A) Body weight changes. (B) RSV lung viral titers. (C) Pulmonary inflammation scores. (D) PAS positive mucus production (%). (E) IFN-γ secreting lung cell spots. (F) IL-4 secreting lung cell spots. Cytokine secreting cell spots were determined after stimulation with CD8 T cell epitope F_92-106_ peptide or CD4 T cell epitope G_183-195_. Results are presented as mean ± SEM. Statistical analysis were analyzed by two-way ANOVA in GraphPad Prism; *** *p*<0.001, ** *p*<0.01. ND: Not detected.

After staining lung sections with periodic acid-schiff (PAS), PAS positive area was analyzed to determine mucus production (Fig 5B and 5F). In contrast to the FI-RSV-A group that showed high PAS staining area, the FI-RSV group did not exhibit PAS positive area before or after challenge indicating no detectable mucus production (Fig 5B and 5F, Fig A panels A-D in S1 File). Furthermore, highest levels of pulmonary inflammation, mucus production, eosinophils, and eotaxin were well correlated with the presence of alum in FI-RSV before (Fig 5G) or after challenge (Fig 5A, 5B and 5H–5J). Whereas, FI-RSV immune mice did not induce siglecF^+^CD11b^+^ eosinophils (Fig 5G–5I) as determined by flow cytometry analysis before or after challenge and eotaxin chemokine in lungs (Fig 5J). Interestingly, live RSV reinfections induced more eosinophils in the airway fluids compared to FI-RSV immunization (live RSV vs. FI-RSV, Fig 5I). Next, we determined the effects of a lower dose of alum in FI-RSV vaccines on inducing protection and disease (Fig 6). Interestingly, a low dose alum (FI-RSV-A 5μg) immune mice displayed less weight loss and suppressed mucus production as well as cleared lung viral loads (Fig 6A, 6B and 6D). However, pulmonary inflammation scores were only slightly lower in the low alum-FI-RSV group without a statistical difference (Fig 6C). Therefore, these results suggest that inactivation of RSV may not be the major factor in inducing vaccine-enhanced RSV disease and that alum adjuvant in FI-RSV vaccine formulations appears to be largely responsible for inducing host immune responses of severe lung inflammation through the mucus production and eosinophilia upon RSV infection.

### Alum in FI-RSV induces Th2 cytokines and IL-4 secreting cell responses in lungs

**Fig 7:**
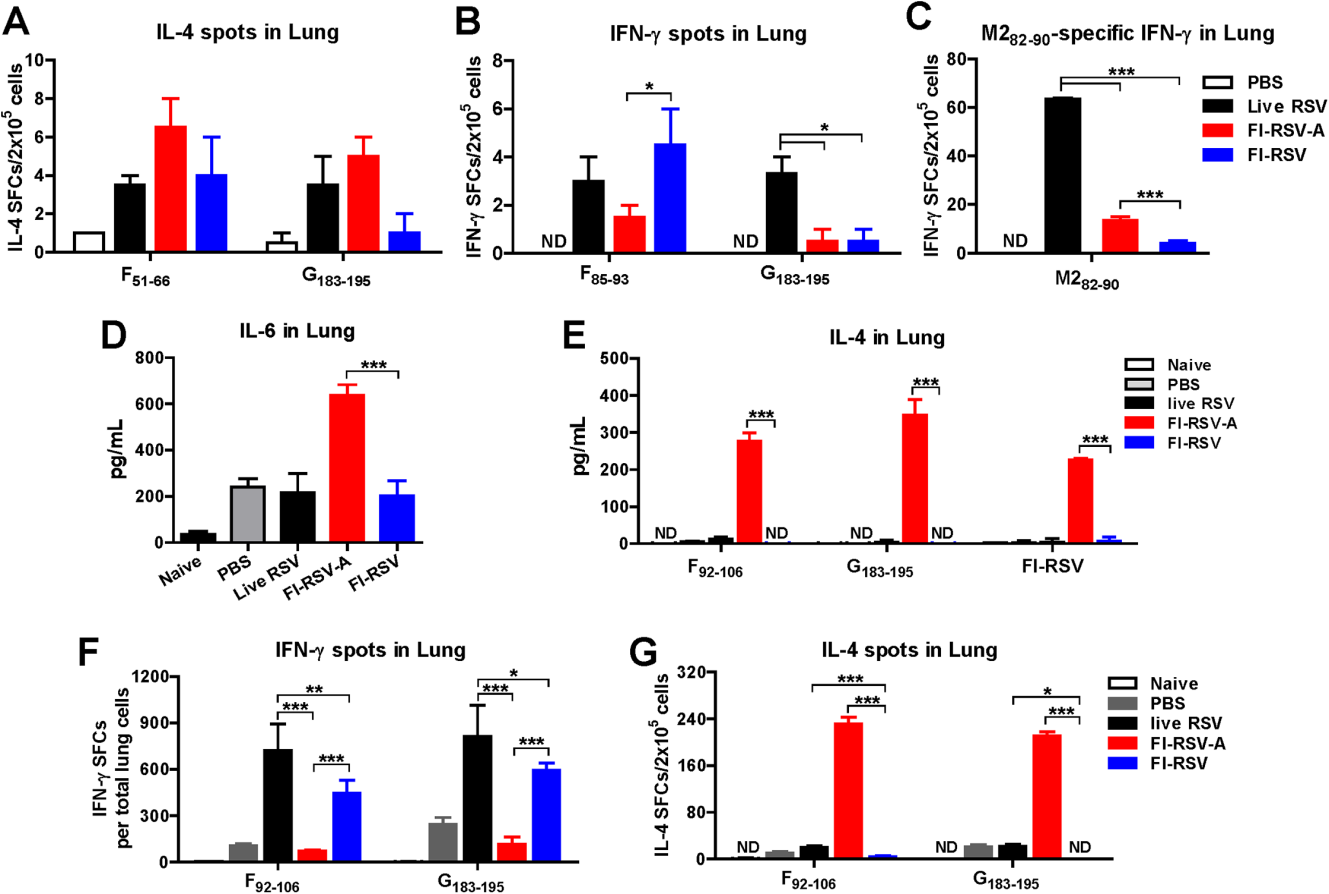
Alum in FI-RSV contributes to inducing Th2 cytokines in the lung. Cytokine levels and cytokine secreting cell responses were determined before (A-C) or after challenge (D-G). (A-C) Cytokine secreting lung cell spots were determined at day 9 post inoculation (live RSV) or post immunization (FI-RSV-A, FI-RSV) before challenge. (A) The numbers of IL-4 secreting lung cell spots specific for F_51-66_ and G_183-195_ peptides at 7 d.p.i.. (Band C) The numbers of IFN-γ secreting lung cell spots specific for F_85-96_, G_183-195_, and M2_82-90_ peptides at 7 d.p.i.. The spots of IFN-γ and IL-4 secreting cells in lungs were detected by stimulation with RSV specific peptides for 36 h using ELISpot analysis. (D) IL-6 in lung homogenates collected at 5 d.p.c.. (E-G) Lung cells were cultured under stimulation with RSV F, G peptide, or inactive RSV (FI-RSV). (E) IL-4 in lungs. The levels of IL-4 were measured in the supernatants harvested after 72 h. (F) IFN-γ secreting lung cell spots. (G) IL-4 secreting lung cell spots. Results are presented as means ± SEM (n = 5). Statistical significances were analyzed by two-way ANOVA in GraphPad Prism; *** *p*<0.001, * *p*<0.05, ND: Not detected.

We determined Th2-biased immune responses by FI-RSV-A before challenge (Fig 7A–7C). Live RSV immune mice induced higher levels of IFN-γ secreting cells by stimulation with G_183-195_ or M2_82-90_ peptide (Fig 7B and 7C) in the lung 7 d.p.i. compared to FI-RSV-A immune mice displaying high levels of IL-4 secreting cell spots upon stimulation with F_51-66_ or G_183-195_ (Fig 7A). In contrast to the FI-RSV-A group, the FI-RSV group showed high levels of F peptide (but not G peptide) specific IFN-γ secreting cell spots and low levels of IL-4 producing cell spots (Fig 7A and 7B). Interestingly, a low alum (5 μg) dose in FI-RSV resulted in significant reductions in IL-4 secreting cell spots and an increase in F_92-106_-specific IFN-γ secreting cell responses compared to high dose alum FI-RSV immune mice (FI-RSV-A 20 μg) (Fig 6E and 6F).

After challenge, we also examined the production of Th1 and Th2 cytokines in the lung extracts collected at day 5 d.p.c. (Fig 7). The FI-RSV-A group was found to be responsible for inducing significantly high levels of IL-6 (Fig 7D) and IL-4 (Fig 7E) proinflammatory cytokines in lungs whereas FI-RSV or live RSV immune mice did not induce IL-4 and IL-6 cytokines in lung extracts. In addition, highest levels of IL-4 secreting cell spots were detected in the lung (Fig 7G) from the FI-RSV-A group but not from FI-RSV or live RSV re-infection mice upon *in vitro* stimulation with peptide F_92-106_ or G_183-195_ (Fig 7G). In contrast, higher numbers of IFN-γ secreting cells in response to RSV F or G peptide stimulation were observed in the lung from the live RSV reinfection and FI-RSV groups (live RSV, FI-RSV, Fig 7F) compared to those in the FI-RSV-A and naïve groups (FI-RSV-A, PBS, Fig 7F). Therefore, our results suggest that alum in FI-RSV induces predominantly Th2-skewed immune responses through excessive production of IL-6 and IL-4 cytokines as well as IL-4 secreting lung cells.

### Alum in FI-RSV contributes to inducing pulmonary IFN-γ^-^IL-4^+^ and IFN-γ^-^TNF-α^+^ memory CD4^+^ T cells

**Fig 8:**
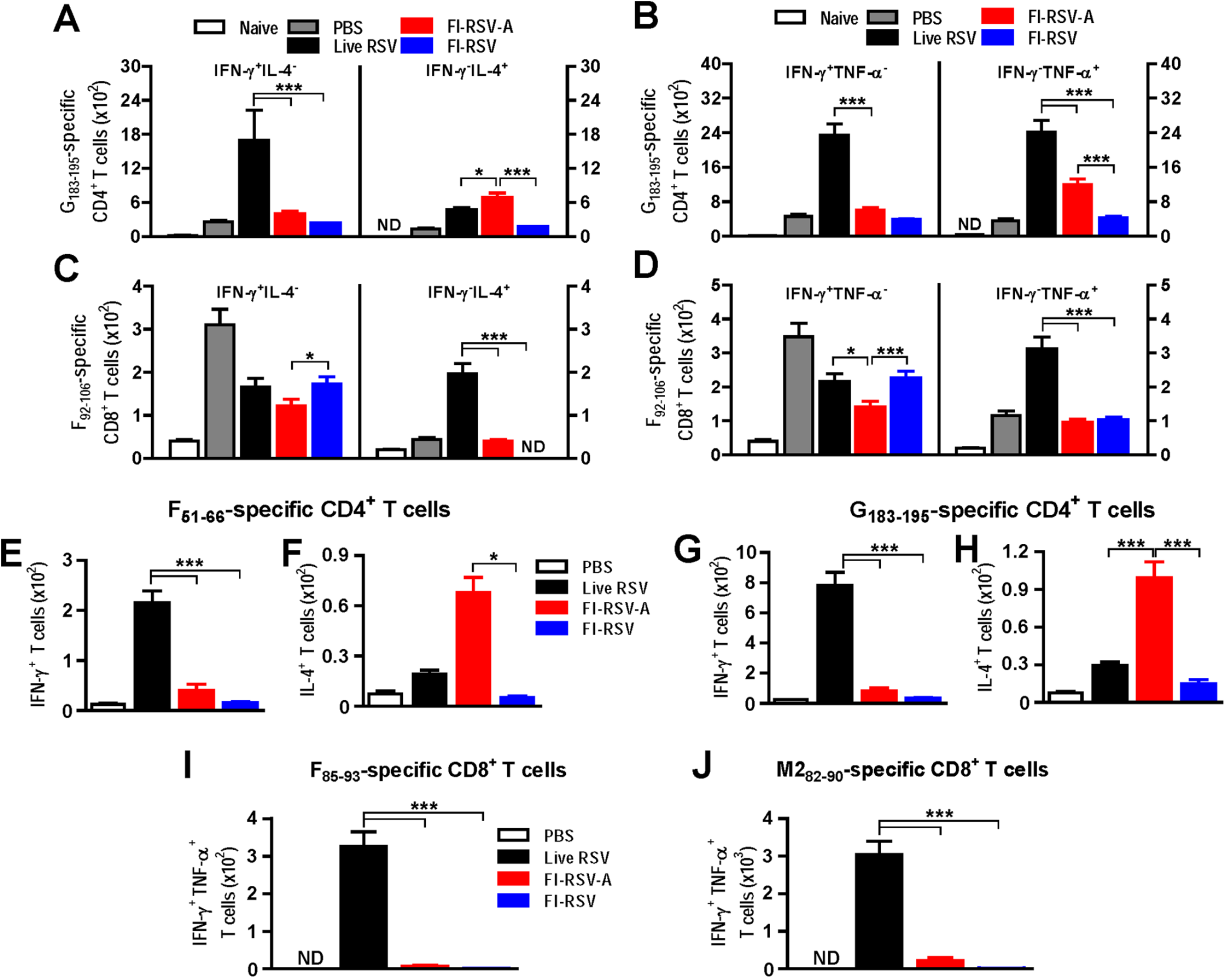
Alum in FI-RSV enhances IFN-γ^-^IL-4^+^and IFN-γ^-^TNF-α^+^ CD4 T cells but inhibits IFN-γ^+^ CD8^+^ T cell responses. Lung cells isolated from mice (n = 5 per group) at 5 d.p.c. (A-D: after challenge) or at 7 d.p.i. (E-I: before challenge) were *in vitro* stimulated with RSV peptides F_92-106_, F_85-90_, M2_82-90_, G_183-195_, or F_51-66_ to determine the effector CD8^+^ and CD4^+^ T cell responses by intracellular cytokine staining flow cytometry. (A and B) G_183-195_-specific CD4^+^ T cells secreting Th1 or Th2 cytokines (IFN-γ^+^IL-4^-^, IFN-γ^-^IL-4^+^, IFN-γ^+^TNF-α^-^, IFN-γ^-^TNF-α^+^) at 5 d.p.c.. (C and D) RSV F_92-106_-specific effector CD8^+^ T cells secreting cytokines (IFN-γ^+^IL-4^-^, IFN-γ^-^IL-4^+^, IFN-γ^+^TNF-α^-^, IFN-γ^-^TNF-α^+^) at 5 d.p.c.. (E and F) F_51-66_-specific CD4^+^ T cells secreting IFN-γ or IL-4 cytokines at 7 d.p.i.. (G and H) G_183-195_-specific CD4^+^ T cells secreting Th1 or Th2 cytokines at 7 d.p.i.. (I and J) RSV F_85-90_ or M2_82-90_-specific effector CD8^+^ T cells secreting IFN-γ^+^ cytokines at 7 d.p.i.. Results are presented as mean ± SEM (n = 5). Statistical analysis were performed by two-way ANOVA in GraphPad Prism; *** *p*<0.001, * *p*<0.05. ND: Not detected.

Using intracellular cytokine staining flow cytometry, we further examined the phenotypes of pulmonary effector T cells producing IL-4, IFN-γ, and TNF-α cytokines upon *in vitro* stimulation with RSV F or G peptide (Fig 8, Fig B in S1 File). FI-RSV-A immune mice showed approximately 5 fold higher numbers of IFN-γ^-^IL-4^+^ and IFN-γ^-^TNF-α^+^ CD4^+^ T cells from lung cell cultures with RSV F_92-106_ peptide stimulation than FI-RSV alone immune mice (*p*<0.001, Fig B panels A-B in S1 File). Also, 3–4 fold higher IFN-γ^-^IL-4^+^ and IFN-γ^-^TNF-α^+^ lung CD4^+^ T cells specific for RSV G_183-195_ peptide were observed in FI-RSV-A immune mice compared to those in FI-RSV immune mice (*p*<0.001 between FI-RSV-A and FI-RSV, Fig 8A and 8B). In contrast, FI-RSV immune mice did not induce IFN-γ^-^IL-4^+^ and IFN-γ^-^TNF-α^+^ CD4^+^ T cells in the lung upon RSV peptide stimulation (Fig 8A and 8B, Fig B panels A-B in S1 File). The live RSV group showed highest levels of IFN-γ^+^IL-4^-^, IFN-γ^+^TNF-α ^-^, and IFN-γ^-^TNF-α^+^ lung CD4^+^ T cells (Fig 8A and 8B, Fig B panels A-B in S1 File).

Before challenge infection with RSV, we also examined the effector CD4 and CD8 T cell responses upon *in vitro* stimulation of various RSV specific peptides (F_51-66_, G_183-195_ as CD4 T cell epitope, F_85-93_ and M2_82-90_ as CD8 T cell epitope) to better understand the effector T cell responses (Fig 8E–8J). Live RSV immune mice induced high levels of IFN-γ secreting CD4 T cells specific for F_51-66_ and G_183-195_ peptides as well as CD8 T cells specific for F_85-93_ and M2_82-90_ peptides compared to those of other groups. In contrast to the live RSV group, FI-RSV-A immune mice exhibited significantly higher levels of IL-4 secreting CD4 T cells specific for F_51-66_ and G_183-195_ peptides. Also, the FI-RSV-A group showed lower levels of RSV-specific IFN-γ secreting CD4 T cells with F_51-66_ and G_183-195_ peptide stimulation and IFN-γ CD8 T cells with F_85-93_ and M2_82-90_ peptide stimulation (Fig 8E–8J). Taken together, these results suggest that alum adjuvant in the FI-RSV vaccine formulation is mainly contributing to the induction of pulmonary IL-4^+^ and TNF-α^+^ CD4^+^ T cells post immunization and during RSV infection.

### Alum in FI-RSV vaccines inhibits the induction of effector CD8^+^ T cells

We investigated possible effects of alum adjuvant on inducing lung CD8 T cell responses by using intracellular cytokine staining flow cytometry analysis. Lung cells from FI-RSV-A immune mice displayed lower levels of RSV F_92-109_ or G_183-195_ peptide-stimulated effector CD8^+^ T cells expressing IFN-γ^+^IL-4^-^ (Fig 8C, Fig B panel C in S1 File), IFN-γ^+^TNF-α^-^ (Fig 8D), and IFN-γ^-^IL-4^+^ (Fig B panel C in S1 File) compared to those in the FI-RSV group. Interestingly, A low dose of alum (5 μg) in FI-RSV resulted in significantly enhancing IFN-γ secreting cell spots upon CD8 T cell epitope RSV F_92-108_ peptide stimulation and suppressing IL-4 secreting cell spots compared to those in high dose alum FI-RSV immune mice (Fig 6E and 6F). As expected, live RSV reinfections induced substantial levels of lung CD8 T cells producing IFN-γ^+^, IL-4^+^, and TNF-α^+^ upon RSV peptide stimulation (Fig 8C and 8D). PBS mock mice infected with RSV also induced relatively high numbers of IFN-γ^+^ CD8^+^ T cells in response to F_92-109_ or G_183-195_ peptide stimulation (Fig 8C and 8D, Fig B panels C and D in S1 File). Taken together, these results suggest that alum adjuvant in FI-RSV vaccine formulation diminishes IFN-γ^+^ effector lung CD8^+^ T cell responses after RSV infection.

### Alum in FI-RSV vaccines raises recruitment of plasmacytoid and CD4^+^ dendritic cells

**Fig 9:**
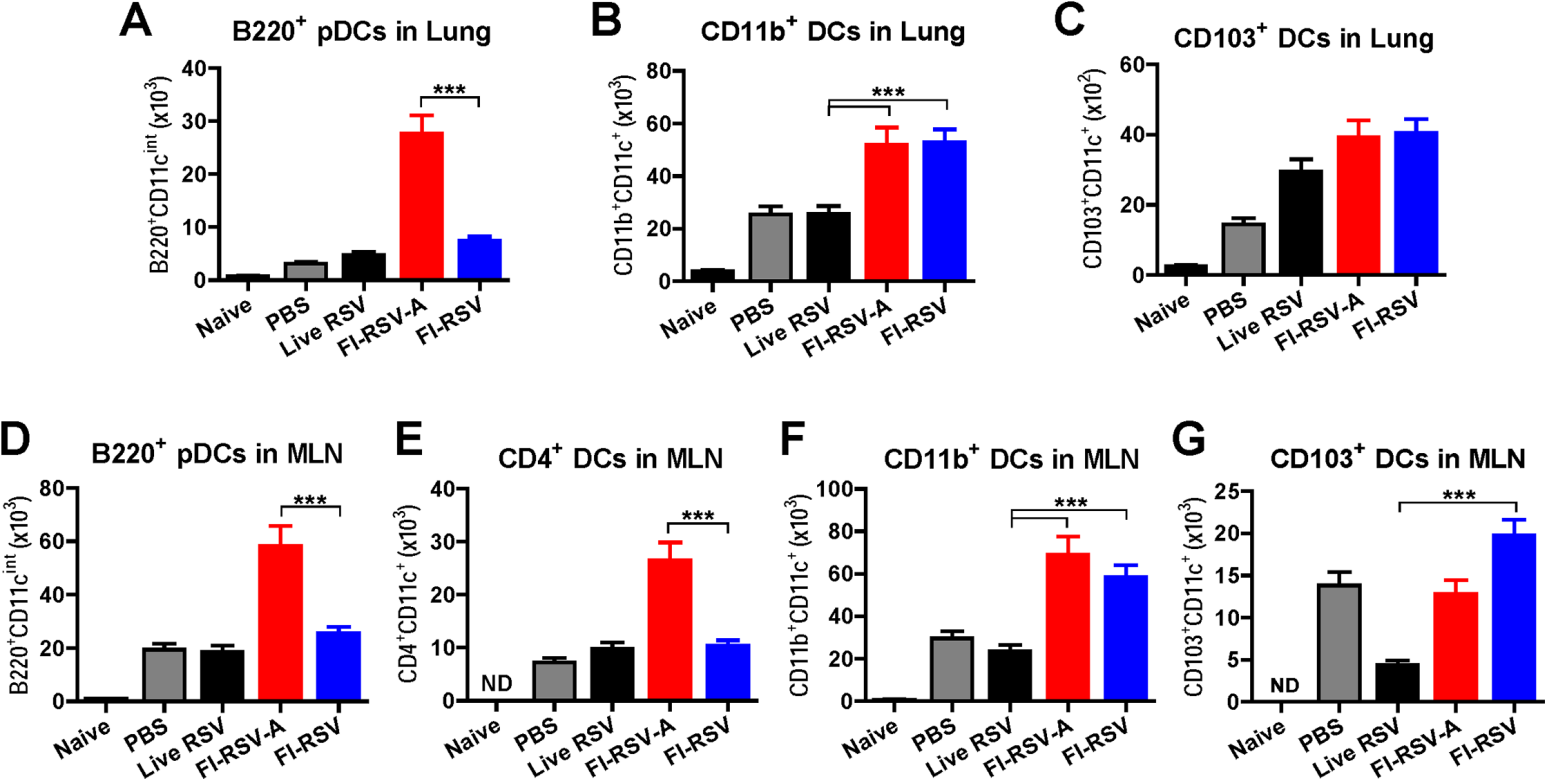
Alum in FI-RSV promotes recruitment of plasmacytoid and CD4^+^ dendritic cells. Lung and MLN cells isolated from mice (n = 5 per group) at 5 d.p.c. were stained with various surface markers and phenotypes of DC subtypes were determined by flow cytometric analysis. (A) Lung B220^+^ pDCs. (B) Lung CD11b^+^ DCs. (C) Lung CD103^+^ DCs. (D) B220^+^ pDCs in MLN. The plasmacytoid DCs (B220^+^CD11c^int^) were counted from gated CD103^-^CD11b^-^F4/80^-^CD45^+^ cells in lungs and MLN. (E) MLN-resident CD4^+^ DCs were determined from gated CD11c^+^CD11b^-^CD45^+^F4/80^-^. (F and G) The population of CD11b^+^ and CD103^+^ DCs were detected from gated CD11c^+^CD11b^-^CD45^+^F4/80^-^ DCs in lungs and MLN. Results are presented as mean ± SEM (n = 5). Statistical analysis was performed by one-way ANOVA in GraphPad Prism; *** *p*<0.001. ND: Not detected.

DCs play a critical role in modulating T cell immunity by producing Th1 or Th2 cytokines upon antigen uptake and presentation [48]. At day 5 post infection, we analyzed different subsets of infiltrating DCs into lungs and mediastinal lymph nodes (MLN) using flow cytometry analysis (Fig 9). The FI-RSV alum group showed highest cell numbers of B220^+^ pDCs (B220^+^CD11c^int^F4/80^-^CD45^+^) in the lung (FI-RSV-A, Fig 9A). In addition, alum adjuvanted FI-RSV immune mice displayed significantly higher numbers of pDCs and CD4^+^ DCs (CD4^+^CD11c^+^B220^-^CD103^-^F4/80^-^CD45^+^) in MLN than those in FI-RSV alone immune mice (Fig 9D and 9E). FI-RSV alone immune mice showed low levels of pDCs and CD4^+^ DCs, which is similar to those in PBS control infection or live RSV re-infected mice (Fig 9A, 9D and 9E). CD11b^+^ (CD11b^+^CD11c^+^F4/80^-^CD45^+^) were observed at higher levels in the lung and MLN from FI-RSV immune mice regardless of alum adjuvant compared to those in live RSV reinfection or naïve mice (Fig 9B and 9F). High levels of CD103^+^ (CD103^+^CD11c^+^F4/80^-^CD45^+^) DCs were recruited into the lungs from live RSV, FI-RSV-A, FI-RSV immune mice compared to those in PBS control mice with RSV infection (Fig 9C). Mice with live RSV reinfection showed lowest recruitment levels of CD103^+^ DCs into MLN compared to other groups of immune mice (Fig 9G). Taken together, these results suggest that alum adjuvant in the FI-RSV vaccine formulation plays a role in recruiting pDCs in lungs and MLN, and CD4^+^ DCs in MLN.

## Discussion

In comparison with live RSV intranasal reinfections, we investigated the possible roles of alum adjuvant in the protection and lung inflammatory disease after FI-RSV intranasal immunization and RSV challenge. FI-RSV alone without alum was found to be weakly immunogenic, inducing low levels of RSV specific antibodies and neutralizing activity. Alum adjuvant significantly increased the levels of IgG1 and IgG2a antibodies specific for RSV F and G proteins. As expected, live RSV is the most effective platform for inducing RSV specific antibodies, in particular IgG2a isotype antibodies. Thus, a replicating property of live RSV seems to be an important factor for enhancing humoral immune responses in addition to the activation of innate immune receptors via intact viral antigens and RNAs. Live RSV, FI-RSV-A, and FI-RSV all showed more antibodies specific for RSV F protein than those for RSV G protein, indicating that F is more immunogenic than G protein. Inclusion of alum adjuvant in FI-RSV vaccines significantly increased levels of RSV neutralizing activity, plasma cells, memory B cells, and germinal center B cells, contributing to clearing lung viral loads. As expected, this study provides further evidence that alum adjuvant was required to enhance immunogenicity and protective efficacy of FI-RSV. The effective clearance of lung RSV loads by FI-RSV alum immunization is consistent with the results in previous studies [39, 49, 50].

Despite the fact that live RSV re-infection was highly effective in clearing lung viral loads, body weight loss was substantial in mice with prior RSV reinfections, which was similar to naïve mice displaying highest lung viral loads. Consistently, FI-RSV-A immune mice showed more weight loss than FI-RSV immune mice after RSV challenge infection. There is no good correlation between RSV lung viral loads and RSV disease in mice since RSV disease of body weight loss was observed regardless of lung viral loads, implicating a limitation of murine models. Nonetheless, results in this study provide convincing evidence that alum adjuvant in the FI-RSV vaccine formulation is contributing to the induction of cellular immune responses associated with FI-RSV vaccine-enhanced respiratory disease.

Histopathology is an important parameter for assessing pulmonary RSV disease. Addition of alum adjuvant to FI-RSV was found to exacerbate pulmonary histopathology as evidenced by highest inflammation scores around the airways, interstitial spaces, and blood vessels. Severe pulmonary inflammation in FI-RSV alum immune mice showed correlations with high levels of infiltrating immune cells and granulocytes, particularly eosinophils, mucus and eotaxin production as well as IL-6 and IL-4 inflammatory cytokines in the lung and BAL fluids of alum-adjuvanted FI-RSV (FI-RSV-A) immune mice, which were not observed in mice with inactivated RSV (FI-RSV) alone immune mice. These results in this study are consistent with previous studies reporting that Th2 cytokines and chemokines were associated with pulmonary eosinophilia and RSV vaccine-enhanced disease [12, 51, 52]. Live RSV re-infection also resulted in substantial degrees of lung inflammatory histopathology, which is similar to FI-RSV alum immunization but more than non-adjuvanted inactivated RSV alone. Thus, simply inactivating RSV would not be a major factor for inducing RSV disease in mice. This is also true that mice immunized with recombinant vaccinia virus expressing RSV G displayed severe lung eosinophilia and weight loss after RSV challenge [53]. Interestingly, co-immunization of UV-inactivated RSV with poly IC, Toll-like receptor (TLR) 3 agonist, or lipopolysaccharide, a TLR4 agonist was shown to diminish UV-inactivated RSV vaccine-enhanced lung inflammation [34]. Further studies are needed for better understanding the adjuvant effects of TLR agonists on improving RSV vaccine safety.

A previous study of antibody-mediated T cell depletion experiments indicated that T cells play a role in the progress of RSV disease [54]. However, what types of effector T cells contributing to disease have not been completely defined yet after alum-adjuvanted FI-RSV vaccination. Interestingly, intracellular staining flow cytometric analysis in this study found that alum adjuvanted FI-RSV immunization induced polarized CD4 Th2 effector cells upon *in vitro* stimulation with F_92-106_, F_51-66_, F_85-93_, and G_183-195_ peptides. In other words, F_92-106_-stimulated IFN-γ^-^IL4^+^ and IFN-γ^-^TNF-α^+^ Th2 CD4 T cells as well as G_183-195_-specific IFN-γ^-^IL-4^+^ Th2 CD4 T cells were observed at significantly higher levels in the lungs of alum-adjuvanted FI-RSV immune mice than those in FI-RSV alone immune mice without alum adjuvant. It is interesting to note that high levels of IFN-γ^-^IL-4^+^ and IFN-γ^-^TNF-α^+^ CD4 T cells in response to CD8 T cell epitope F_92-106_ peptide stimulation were observed in the FI-RSV-A group as determined by intracellular cytokine staining (Fig A panels A-B in S1 File). It might be possible to induce CD4 T cells from alum-adjuvanted FI-RSV-A immune mice through bystander stimulation with 15-mer F peptides *in vitro*. In contrast, the induction of CD8 T cells in response to CD4 T cell epitope G_183-195_ peptide stimulation was inhibithed in FI-RSV-A immune mice compared to FI-RSV mice without alum (Fig B panels C-D in S1 File). It is still unclear how the G_183-195_ peptide known to be a CD4 T cell epitope can stimulate CD8 T cells. Th2 type CD4 T cells responding to both RSV F and G peptide stimulation might be due to bystand stimulation through high levels of IL-4 cytokine and contributing to alum-adjuvanted FI-RSV vaccine-enhanced pulmonary histopathology. Whereas live RSV re-infection induced high levels of IFN-γ^+^IL-4^-^ and IFN-γ^+^TNF-α^-^ Th1 CD4 T cells as well as IFN-γ^-^TNF-α^+^ CD4 T cells in response to RSV F and G peptide stimulation. High levels of Th1 type CD4 T cells are also considered to be involved in RSV disease as evidenced by weight loss and pulmonary histopathology in the live RSV group. FI-RSV alone immune mice showed low levels of cytokine-secreting CD4^+^ T cells, which might be contributing to less pulmonary inflammation. Therefore, this study provides evidence that high levels of IFN-γ^-^IL-4^+^ and IFN-γ^-^TNF-α^+^ CD4 T cells might be associated with pulmonary histopathology, which are highly induced by alum adjuvant in FI-RSV.

Mice with RSV re-infection induced high levels of IFN-γ^+^IL-4^-^, IFN-γ^-^IL-4^+^, IFN-γ^-^TNF-α^+^, and IFN-γ^+^TNF-α^-^ CD8 T cells upon *in vitro* stimulation with RSV F and G peptides in contrast to mice with alum-adjuvanted FI-RSV that showed very low levels of effector CD8 T cells. Therefore, RSV re-infection can induce RSV specific effector CD4 and CD8 T cells producing both Th1 and Th2 type cytokines whereas alum-adjuvanted FI-RSV prefers to stimulate IL-4^+^ or TNF-α^+^ Th2 CD4 T cells but not effector CD8 T cells. Whereas primary RSV infection (naïve mice with RSV) or FI-RSV alone immune mice are likely to induce RSV F and G specific CD8 T cells expressing IFN-γ^+^ but not IL-4^+^ CD4 T cells. Experimental evidence in this study suggests that alum adjuvant in FI-RSV suppresses the induction of RSV specific IFN-γ^+^ CD8 T cells. RSV specific effector T cell responses are differentially induced depending on primary RSV infection of naïve mice (PBS mock control), RSV re-infection, alum-adjuvanted FI-RSV, or inactivated RSV immunization.

Both FI-RSV with and without alum groups were significantly lower in the levels of IFNγ producing CD4 and CD8 T cells, in particular F and M2 specific IFN-γ CD8 T cells (Fig 8, Fig B panels C-D in S1 File). Alum adjuvant in FI-RSV appeared to suppress the induction of IFN-γ CD8 T cells compared to FI-RSV without alum. Since there was no significant difference in lung viral loads between the live RSV and FI-RSV plus alum groups, CD8 T cells might not be required for clearing lung viral loads of RSV. In line with this notion, depletion of CD8 T cells after primary RSV infection of mice was reported not to affect clearing lung viral loads during re-infection with RSV [34]. Instead, CD8 T cells are likely to play a role in modulating or suppressing RSV vaccine-enhanced disease. However, high levels of both IFN-γ CD4 and CD8 T cells might cause pulmonary pathology. These results in this study are consistent with previous reports that RSV M2 peptide specific IFN-γ CD8 T cells play a significant role in causing CD8 T cell-mediated immunopathology including weight loss in mice upon RSV challenge [55, 56]. In this regard, it is worth noting that RSV re-infection resulted in enhanced levels of both effector CD4 and CD8 T cells secreting cytokines, which might be responsible for pulmonary inflammation and weight loss despite effective control of lung viral loads. Therefore, in addition to RSV neutralizing activity essential for lung viral clearance, induction of regulated and restricted T cell responses preferentially CD8 T cells as well as preventing excessive CD4 T cell responses would be important for RSV protection without disease.

It is considered that mobilization of DCs would provide an important immune regulator for modulating innate microenvironment and adaptive immune T cell responses as well as for pulmonary histopathology. In particular, different subsets of DCs were suggested to play a critical role in inducing RSV-specific adaptive immune responses [57]. However, the possible roles of alum adjuvant in modulating DC subsets as well as in protection and RSV disease are not well known. FI-RSV alum immune mice displayed approximately 3-fold higher B220^+^ pDC populations (B220^+^CD11c^int^F4/80^-^CD45^+^) in the lung and MLN compared to those in inactivated RSV alone or live RSV immune mice. Therefore, recruitment of high levels of pDCs in both lungs and MLN is likely to be associated with inflammatory pulmonary RSV disease in addition to IL-4 and IL-6 proinflammatory cytokines as well as mostly CD4 T cells secreting IL-4 or TNF-α. Consistent with possible roles of pDCs in RSV disease in FI-RSV alum immune mice, pDCs were shown to increase mortality during influenza virus infections by inhibiting CD8 T cell responses [33]. Also, human pDCs and conventional DCs were demonstrated to induce Th2 responses via polarizing cytokines, costimulatory molecules, and cell surface receptors [58–60]. Detailed analysis of DC subsets in this study also found that alum adjuvant induced high levels of CD4^+^ DCs in MLN from FI-RSV alum immune mice. In support of possible roles of CD4^+^ DCs in inducing IL-4 cytokine and IL-4^+^ CD4 T cells in FI-RSV alum immune mice, CD4^+^ DCs were reported to enhance Th2 differentiation by inhibiting IL-12 and IFN-γ production [28, 61]. To obtain an insight into differential host responses by alum adjuvant in FI-RSV, bone marrow derived DCs and macrophages were incubated with live RSV, FI-RSV plus alum, and inactivated RSV (FI-RSV) alone. The IL-6 and TNF-α cytokines were detected in culture supernatants at significantly higher levels in stimulation with live RSV or alum-adjuvanted FI-RSV compared to those with FI-RSV alone (Fig C panels A-B in S1 File). Thus, alum adjuvant in the FI-RSV formulation was found to significantly enhance the activation of dendritic cells to secrete IL-6 and TNF-α inflammatory cytokines *in vitro* (Fig C panels A-B in S1 File). IL-6 is known to inhibit the induction of Th1 immune responses [62]. Thus, the possible modulation of alum adjuvant in activating DCs and macrophage cells might be supportive of high levels of TNF-α^+^ CD4 T cells and Th2 (IL-4, IL-6) cytokines in alum-adjuvanted FI-RSV immune mice.

In summary, live RSV re-infection, alum-adjuvanted FI-RSV, and FI-RSV without alum significantly influence the induction of Th1 and Th2 cytokines by differential CD4 and CD8 T cell responses as well as the mobilization of multiple DC subsets and eosinophils. Both innate and adaptive host immune components play significant roles in RSV protection and disease in mice. In particular, alum adjuvant in FI-RSV vaccine formulations has considerable impacts on increasing immunogenicity and virus clearance as well as Th2 type CD4 T cells, proinflammatory cytokines, eosinophilia, mucus production, B220^+^ pDCs, and CD4^+^ DCs, and inhibition of effector CD8 T cells. Results in this study suggest that induction of a balanced immune response with appropriate CD4 and CD8 T cells, and appropriately regulated innate pulmonary DC subsets in addition to RSV neutralizing activity would be required for a safe and effective RSV vaccine. Further studies are required to delineate possible roles of respiratory DC subsets in the RSV protection and pulmonary histopathology with different RSV vaccine platforms.

## Supporting Information

S1 File Supplementary Figures A-C. (DOCX)
