# Supplementary material for: Alum Adjuvant Enhances Protection against Respiratory Syncytial Virus but Exacerbates Pulmonary Inflammation by Modulating Multiple Innate and Adaptive Immune Cells
Source: PLoS One. 2015 Oct 15;10(10):e0139916. doi: 10.1371/journal.pone.0139916 (PMC4607166; doi:10.1371/journal.pone.0139916)
Supplement: S1 File — (DOCX) [file pone.0139916.s001.docx]

**Supporting Information (SI)**


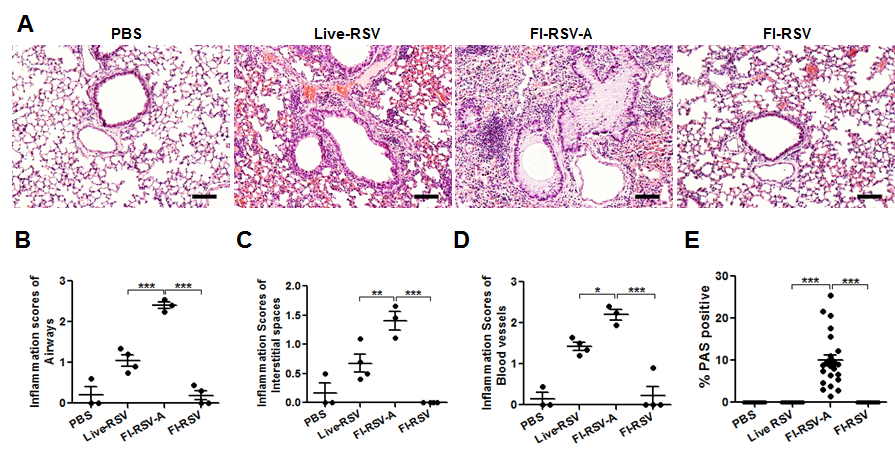


**Fig A. Lung histology of mice with live RSV re-infection or FI-RSV vaccination before challenge.**

Lung tissues were collected from individual mice (n=3 or 4 per groups) at 7 days post immunization and stained with Hematoxylin and Eosin (H&E) and Periodic-Schiff (PAS) to assess peribronchiolar, alveolar pneumonia, and mucus production. (A) Photomicrographs of H&E. H&E stained tissue sections were scored in the airways (B), interstitial spaces (C), and blood vessels (D). (E) PAS positive airway mucus production. Results are presented as mean ± SEM. Statistical analysis were analyzed by one-way ANOVA and Tukey’s multiple comparison test in GraphPad Prism; *** *p*<0.001, ** *p*<0.01, * *p*<0.05.


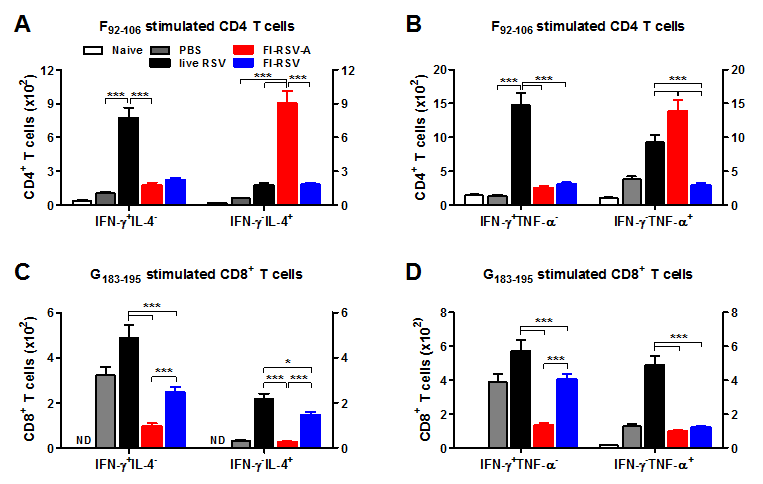


**Fig B. Atypical effector CD4 and CD8 T cell responses in response to *in vitro* stimulation with RSV F_92-106_ and G_183-195_** **peptide.**

Lung cells collected at 5 d.p.c. were *in vitro* stimulated with RSV F_92-106_ and G_183-195_ peptide to determine the effector CD8^+^ and CD4^+^ T cells by intracellular cytokine staining flow cytometry. (A and B) F_92-106_ peptide-stimulated IFN-γ (IFN-γ^+^IL-4^-^, IFN-γ^+^TNF-α^-^), IL-4 (IFN-γ^-^IL-4^+^), or TNF-α (IFN-γ^-^TNF-α^+^) secreting CD4^+^ T cells. (C and D) RSV G_183-195_ peptide- stimulated effector CD8^+^ T cells secreting cytokines (IFN-γ^+^IL-4^-^, IFN-γ^-^IL-4^+^, IFN-γ^+^TNF-α^-^, IFN-γ^-^TNF-α^+^). Results are presented as mean ± SEM (n=5). Statistical analysis was carried out by two-way ANOVA in GraphPad; *** *p*<0.001, * *p*<0.05. ND: non detection.


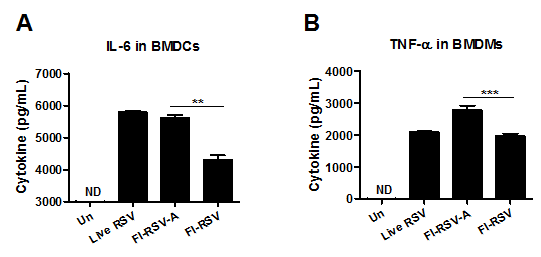


**Fig C. Alum adjuvant stimulates bone marrow derived dendritic and macrophage cells to secrete inflammatory cytokines.**

Bone marrow-derived dendritic (BMDCs) and macrophages (BMDMs) were obtained from BALB/c. BM cells were harvested from femur and tibia and red blood cells (RBC) were removed by RBC lysis buffer (Sigma Aldrich, St. Louis, MO). For preparation of BMDCs and BMDMs, the BM cells were cultured in completed RPMI 1640 or DMEM medium containing 10 ng/ml mouse recombinant GM-CSF (Invitrogen) or 25 ng/ml M-CSF (R&D Systems, Minneapolis, MN) respectively for 6 to 10 days at 37°C in a 5% CO_2_. BMDCs and BMDMs were stimulated with 10 μg of purified RSV, FI-RSV with (FI-RSV-A) or without (FI-RSV) alum for 20 h. The production of TNF-α and IL-6 cytokines from stimulated supernatants were measured by ELISA. (A) IL-6 production in BMDCs. (B) TNF-α production in BMDMs. Media control was not detected (not shown). Data shown are representative of three independent experiments. Results are given as mean ± SEM and statistical significance was performed by two-way ANOVA in GraphPad Prism; *** *p*<0.001, ** *p*<0.01.
